# Neoadjuvant immunochemoradiotherapy with nivolumab, paclitaxel, and cisplatin followed by esophagectomy for locally advanced esophageal squamous cell carcinoma

**DOI:** 10.1038/s41416-026-03349-6

**Published:** 2026-03-20

**Authors:** Ta-Chen Huang, Jhe-Cyuan Guo, Chia-Chi Lin, Hung-Yang Kuo, Chien-Huai Chuang, Jason, Chia-Hsien Cheng, Feng-Ming Hsu, Jang-Ming Lee, Pei-Ming Huang, Cher-Wei Liang, Yu-I Li, Chih-Hung Hsu

**Affiliations:** 1 Graduate Institute of Oncology, National Taiwan University College of Medicine, Taipei, Taiwan; 2 Department of Oncology, National Taiwan University Hospital, Taipei, Taiwan; 3 National Taiwan University Cancer Center, National Taiwan University College of Medicine, Taipei, Taiwan; 4 Department of Surgery, National Taiwan University Hospital, Taipei, Taiwan; 5 Department of Pathology, Fu-Jen Catholic University Hospital, New Taipei City, Taiwan

**Keywords:** Oesophageal cancer, Oesophageal cancer

## Abstract

**Background:**

Nivolumab is effective in treating patients with esophageal squamous cell carcinoma (ESCC). The efficacy of nivolumab in combination with neoadjuvant chemoradiotherapy (CRT) remains unclear.

**Methods:**

In this phase II trial with Simon’s 2-stage design, neoadjuvant nivolumab, paclitaxel, and cisplatin were administered with radiotherapy followed by esophagectomy to patients with locally advanced ESCC. The primary endpoint was pathological complete response (pCR). The secondary endpoints were feasibility, safety, recurrence-free survival (RFS), progression-free survival (PFS), and overall survival (OS).

**Results:**

A total of 17 patients were enrolled in stage I. Fourteen patients received esophagectomy, with pCR achieved in 4, below the criterion for continuing to stage II. All grade and ≥grade 3 treatment emergent adverse events (TEAEs) occurred in 15 and 4 patients, respectively. Immune-related TEAEs occurred in 7; none were ≥ grade 3. The median durations of RFS, PFS, and OS were 8, 12, and 25 months, respectively. Patients with high expression of PD-L1 had higher pCR rate (100% vs 18%, *P* = 0.019).

**Conclusions:**

Nivolumab in combination with neoadjuvant CRT is safe for patients with locally advanced ESCC, and may be beneficial in those with high PD-L1 expression.

**Clinical trial registration:**

NCT05130684.

## Background

Esophageal cancer (EC) is a major malignant disease worldwide. According to the GLOBOCAN 2020 database, approximately 604,100 new cases of EC and 544,100 EC-associated deaths were reported, making EC the 9th most common type of cancer and the 6th most common cause for cancer-related death worldwide. EC is divided into 2 major histological subtypes: esophageal squamous cell carcinoma (ESCC) and esophageal adenocarcinoma (EAC). ESCC accounts for 85% of the global burden of EC [1], particularly in Asia.

The majority of patients with ESCC present with locally advanced disease, namely stage T3/T4 or N(+) as per the Tumor, node, metastasis staging system, at diagnosis. According to 2 large randomized controlled trials, neoadjuvant chemoradiotherapy (CRT) followed by surgery is currently the global standard of care for patients with locally advanced EC, particularly for those with ESCC. In the CROSS trial, which included European patients with EAC and ESCC, neoadjuvant CRT was found to have a statistically significant survival benefit when administered as a combination of paclitaxel and carboplatin on a weekly basis [2]. Subgroup analysis revealed that the survival benefit conferred by this combination was more pronounced in patients with ESCC than in those with EAC. In another randomized trial, Yang et al. [3] enroled patients with ESCC from multiple centres in China. They reported that administering neoadjuvant CRT as a combination of vinorelbine, a microtubule-acting agent, and cisplatin resulted in an increased pathological complete response (pCR) rate and a statistically significant survival benefit in patients with localised ESCC. However, a 10-year follow-up report of the CROSS study revealed that while neoadjuvant CRT reduced the risk of locoregional recurrence, it did not improve the rate of distant metastasis, suggesting that additional systemic therapy combined with locoregional therapy may offer further benefit to improve the outcomes of patients with locally advanced ESCC [4].

Cancer immunotherapy, particularly with the blockade of immune checkpoints such as programmed cell death protein 1 (PD-1), has become a new paradigm of cancer therapy and has transformed the standard of care for multiple types of cancer, including ESCC [5] (https://www.accessdata.fda.gov/drugsatfda_docs/label/2019/125514s065lbl.pdf). Multiple phase III studies have indicated that anti-PD-1 monotherapy is an effective treatment for patients with advanced ESCC who have undergone platinum-based chemotherapy [6–10]. Multiple phase III trials have also revealed that combining anti-PD-1 antibodies with cisplatin-based chemotherapy improves the overall survival (OS) of patients with ESCC who have undergone first-line systemic therapy for a recurrent or metastatic disease [11–18]. In 2021, the CheckMate 577 study demonstrated that adjuvant anti-PD-1 therapy with nivolumab significantly increased the duration of disease-free survival in patients with locoregional EC or gastroesophageal junction cancer who have undergone neoadjuvant CRT and achieved R0 resection but remained with pathologic residual disease [19]. However, as of this report, the benefit of integrating anti-PD-1 immunotherapy into the neoadjuvant CRT regimen of patients with locally advanced ESCC remains unclear.

Administering anti-PD-1 antibodies during an early stage of therapy (neoadjuvant therapy instead of adjuvant therapy) may theoretically benefit patients with cancer. Neoadjuvant therapy aims to improve the surgical outcomes of patients with resectable or potentially resectable tumours by increasing their R0 resection rate and eliminating micrometastasis. A preclinical study of breast cancer demonstrated the advantages of neoadjuvant therapy with immune checkpoint inhibitors (ICIs) over adjuvant ICIs [20]. Generally, the intact lymphatic and vascular systems around tumours facilitate the interaction between tumour cells (TCs) and immune cells (ICs). In addition, the presence of a wider range of tumour neoantigens may induce a stronger systemic antitumor response and earlier immune memory [21, 22]. A randomized phase II trial demonstrated that administering neoadjuvant pembrolizumab to patients with resectable stage III or IV melanoma significantly increased their event-free survival [23]. Additionally, the CheckMate 816 study revealed that integrating nivolumab into the neoadjuvant chemotherapeutic regimen of patients with resectable NSCLC increased their pCR rate and event-free survival [24]. Similarly, the KEYNOTE-671 trial reported that integrating pembrolizumab into the neoadjuvant chemotherapeutic regimen of patients with resectable NSCLC undergoing adjuvant pembrolizumab therapy increased their event-free survival and OS [25]. These promising findings jointly underscore the potential benefit of anti-PD-1 immunotherapy as a neoadjuvant anticancer therapy for patients with other types of cancer, such as ESCC.

In this study, we hypothesised that the integration of nivolumab into neoadjuvant paclitaxel and cisplatin-based CRT followed by esophagectomy is safe and effective for patients with locally advanced ESCC.

## Methods

### Study design

This study was conducted as a single-arm, single-centre, open-label phase II Simon’s 2-stage investigator-initiated trial (ClinicalTrials.gov identifier: NCT05130684). It was designed by lead investigators at National Taiwan University Hospital and was partially funded by ONO Pharmaceutical Company and Bristol Myers Squibb biopharmaceutical company. It was conducted in accordance with the principles of the Declaration of Helsinki and Good Clinical Practice guidelines. The study protocol was approved by the Institutional Research Ethics Committee of National Taiwan University Hospital (approval no. 202005115MIPB). All patients provided written informed consent before their screening and enrolment.

### Patients

Patients aged between 20 and 75 years with histologically confirmed intrathoracic locally advanced ESCC (stage cT3-4aN0 or cT1-3N1-3M0 according to the *Eighth Edition AJCC Cancer Staging Manual*) with a limited tumour burden (longitudinal length ≤8 cm, radial length ≤5 cm) were included in the study. Patients meeting the following criteria were enroled: having an Eastern Cooperative Oncology Group (ECOG) performance score of 0 to 1; having adequate bone marrow, liver, and kidney function; and following effective contraceptive methods. Patients meeting the following criteria were excluded: having esophageal or gastroesophageal junction adenocarcinoma; having synchronous squamous cell carcinoma in the upper aerodigestive tract but not in the esophagus; having a history of thoracic irradiation, systemic chemotherapy, or treatment with anti-PD1, anti-PD-L1, or anti-PD-L2 agents; having an active autoimmune disease or any other conditions requiring long-term systemic corticosteroids or immunosuppressive agents over the past 2 years; or having a history of primary immunodeficiency, active pulmonary tuberculosis, noninfectious pneumonitis, or organ transplantation. Patients with active hepatitis B (HBsAg-reactive) or hepatitis C (anti-HCV-reactive with HCV RNA detected) infection and patients who have received a live vaccine within 30 days of the study were also excluded.

### Patient assessment at baseline

All patients underwent physical examinations and baseline tumour assessments with endoscopic ultrasonography (EUS), bronchoscopy, esophagoscopy, ^18^F-fluorodeoxyglucose positron emission tomography (FDG-PET), and computed tomography (CT) with contrast for the brain, neck, chest, and abdomen areas within 28 days before the initiation of therapy.

### Treatment protocol

A combination of nivolumab and paclitaxel/cisplatin-CRT [26, 27], referred to as neo-NTP-CRT, was administered as a neoadjuvant therapy. This combination consisted of 240 mg of nivolumab (administered intravenously for 30 min on days −14, 1, 15, and 29), 50 mg/m^2^ paclitaxel (administered intravenously for 1 h on days 1, 8, 15, 22, and 29), and 30 mg/m^2^ cisplatin (administered intravenously for 2 h on days 1, 8, 15, 22, and 29), concurrent with radiation at 1.8 Gy/fraction for 5 days a week for a total of 25 fractions (with a total radiation dose of 45 Gy). Radiotherapy was delivered using megavoltage linear accelerators (6 or more megavoltage photons) with multiple fields (2 or 3 fields). A CT simulation was conducted to determine the isodose distribution and obtain a dose-volume histogram. The clinical target covered regions at risk, including primary esophageal tumours and metastatic nodal stations. Radical surgery involving right thoracic minimally invasive esophagectomy with 2- or 3-field lymph node dissection and gastric tube reconstruction was scheduled 6 to 8 weeks after the completion of neo-NTP-CRT. In case a patient did not undergo surgery after their post-CRT response evaluation, a second round of CRT was administered with a cumulative RT dose of 66 Gy.

### Response evaluation and patient follow-up

After 3–4 weeks of neo-NTP-CRT, all patients underwent examinations using EUS, bronchoscopy, FDG-PET, and CT scan for the brain, neck, chest, and abdomen regions to evaluate their clinical response to neo-NTP-CRT. For patients who underwent an esophagectomy, all resected samples, including those of the esophagus and regional lymph nodes, were subjected to a routine pathologic examination. To detect tumour recurrence or progression after treatment, patients were followed up through clinic visits every month in year 1, every 2 months in year 2, and every 3 months from years 3 to 5. This follow-up procedure involved endoscopy every 6 months in the first 2 years and then annually from year 3 to year 5, with CT scans conducted for the brain, neck, chest, and abdomen regions every 3 months in the first 2 years and then every 6 months from years 3 to 5.

### Endpoints

The primary endpoint for this study was pCR, which was defined as the absence of viable cancer cells in the resected samples of the esophagus and regional lymph nodes in routine pathological evaluations. The secondary endpoints were feasibility, safety, toxicity, progression-free survival (PFS), recurrence-free survival (RFS), and OS. Feasibility was defined as the successful completion of neo-NTP-CRT and surgery without a significant treatment delay, defined as more than 19 weeks from the first dose of nivolumab (day −14) to the day of surgery (including 15 weeks of treatment and 4 weeks of flexibility). Safety was defined as the absence of a significant increase in the rate of treatment-related death from the first dose of nivolumab to 30 days after esophagectomy (6.67% vs 2.67% of our historical control). Toxicity was defined as the presence of adverse events, major adverse events, or immune-related adverse events. PFS was defined as the time from registration to the first detection of tumour progression or recurrence or patient death. RFS was defined as the time from curative surgery to the first detection of tumour recurrence or patient death. OS was defined as the time from registration to patient death. Tumour progression and recurrence were defined in accordance with Response Evaluation Criteria in Solid Tumours (RECIST) version 1.1 [28]. Adverse events were graded and recorded throughout the study and during the follow-up period in accordance with National Cancer Institute Common Terminology Criteria for Adverse Events version 5.0.

### Sample size calculation

The required sample size was calculated in accordance with the primary endpoint, namely the rate of pCR in an intent-to-treat population. We hypothesised that the pCR rate of neo-NTP-CRT would increase from 25% (historical control) to 45%, a rate considered to be clinically significant. To comply with Simon’s 2-stage design, with a one-sided alpha value of 0.05 and a power of 80%, 17 patients would be enroled in stage 1. If 5 or more patients achieved pCR, another 24 patients (41 patients in total) would be enroled in stage 2. If a total of 15 or more patients achieved pCR, the null hypothesis would be rejected, and the primary endpoint of the clinical trial would be met.

### Biomarker study

#### Immunohistochemistry

Endoscopic biopsy specimens of pretreated primary esophageal tumours were collected to conduct a biomarker study. Formalin-fixed, paraffin-embedded tissue sections were retrieved from the Pathology Department of National Taiwan University Hospital. A pathologist (Liang) confirmed that all tissue samples contained adequate tumour lesions, representing more than 50% of all samples upon hematoxylin and eosin staining. Tissue slides for immunohistochemical staining were prepared using a previously described method [29]. Samples were blocked with Dual Endogenous Enzyme Block (DakoCytomation, Glostrup, Denmark) before their incubation with primary antibodies against PD-L1 (dilution 1:100, clone SP142; Ventana, AZ, USA), CD20 (clone L26; Zytomed, Berlin, Germany), or CD23 (clone DAK-CD23; Agilent Dako, Santa Clara, CA, USA). Subsequently, the samples were washed and incubated for 30 min with corresponding secondary antibodies (Dako REAL EnVision HRP rabbit/mouse). After washing, the samples were treated with diaminobenzidine and hematoxylin. All samples were independently evaluated by 2 pathologists (Liang and Li). Any discrepancies between pathologists were resolved through discussion until a consensus was reached. The expression levels of PD-L1 on TCs and ICs and the combined positive score (CPS) were independently scored in accordance with established scoring systems [30, 31]. The expression of PD-L1 on TCs was scored as TC0 (<1%), TC1 (≥1% and <5%), TC2 (≥5% and <50%), or TC3 (≥50%) and categorised as either positive (TC1, TC2, and TC3) or negative (TC0). The expression of PD-L1 on ICs was scored as IC0 (<1%), IC1 (≥1% and <5%), IC2 (≥5% and <10%), or IC3 (≥10%) and categorised as either high (IC2 and IC3) or low (IC0 and IC1). The CPS of PD-L1 expression was categorised as either high (CPS ≥ 5) or low (CPS < 5). Tertiary lymphoid structures (TLSs) were classified as immature TLSs with moderate or strong aggregation of CD20 expression without CD23 expression within CD20 aggregations or as mature TLSs with moderate or strong aggregation of CD20 expression with CD23 expression within CD20 aggregations. Patients were divided into the following groups depending on their TLS status: no TLS (absence of immature or mature TLSs in tissue), immature TLS (presence of immature TLSs and absence of mature TLSs in tissue), and mature TLS (presence of mature TLSs in tissue).

#### RNA expression of immune-related genes

Formalin-fixed, paraffin-embedded tissue sections of pretreated primary esophageal tumours were retrieved from the Pathology Department of National Taiwan University Hospital. These samples were subjected to PanCancer Immune Profiling and were analysed using an nCounter Analysis System (NanoString Technologies, Seattle, WA, USA). In addition, their 12-chemokine TLS signature was analysed [32–36].

### Statistical analysis

A database lock was performed on August 18, 2024, with analyses conducted following an intention-to-treat principle. Descriptive statistics were used to account for baseline characteristics, adverse events, safety, feasibility, toxicity, and pCR rate. Kaplan-Meier survival curves were used to estimate survival distributions. As for biomarker research, we used chi-square test to examine the association of TCs, ICs, and CPS of PD-L1 expression and mature TLSs with pCR, and used *t*-test to examine the association between 12-chemokine TLS signature and pCR. A log rank test was used to examine the association of OS with TCs, ICs, and CPS of PD-L1 expression and mature TLSs. Differences in ICs and immune-related functions between patients with and without pCR were examined using a *t*-test. All statistical analyses were conducted using GraphPad Prism version 5.01 (GraphPad Software, San Diego, CA, USA).

## Results

### Study population

Between January and April 2021, a total of 6 patients were enroled in the safety run-in phase of the trial to evaluate the safety and feasibility of the proposed protocol. After the safety of the clinical trial was confirmed, with all 6 patients completing their treatment, another 11 patients were enroled from February 2022 to April 2023 for the first stage of the study. However, the trial did not meet the primary endpoint to enter the second stage; therefore, the enrolment process was discontinued. A total of 17 patients, with a median age of 52 years (range: 44–69 years, 15 men), were enroled. Their baseline characteristics are summarized in Table 1. Of these patients, 9 had an ECOG performance score of 0, whereas 8 had an ECOG performance score of 1. Primary esophageal tumours were located in the upper, middle, and lower thoracic esophagus regions in 4, 7, and 6 patients, respectively, with 15 patients categorised into stage III, 1 patient categorised into stage I, and 1 patient categorised into stage II, as per the *Eighth Edition AJCC Cancer Staging Manual*.

**Table 1:** Baseline characteristics of the patients.

		No.	%
Total patient number		17	100
Age			
	Median (range)	52 (44–69)	
Sex			
	M	15	88.2
	F	2	11.7
ECOG-PS			
	0	9	52.9
	1	8	47.0
Primary tumor location			
	Upper thoracic	4	23.5
	Middle thoracic	7	41.1
	Lower thoracic	6	35.2
Clinical stage*			
	T1N1	1	6
	T1N2	1	6
	T2N1	1	6
	T2N2	1	6
	T3N0	0	
	T3N1	5	29
	T3N2	8	47
	T3N3	0	0
Risk factor exposure			
	Alcohol	16	94.1
	Cigarette	15	88.2
	Betel nut	8	47.0

*ECOG* Eastern Cooperative Oncology Group, *F* female, *M* male, *PS* performance status.

*Clinical stage according to AJCC 8th ed.

### Treatment

#### Neo-NTP-CRT

All patients except 2 received all 4 doses of 240 mg of nivolumab on days −14, 1, 15, and 29. Treatment with nivolumab was stopped twice in 1 patient because he developed grade 2 colitis, and 1 dose of nivolumab was skipped in another patient because he developed grade 2 skin rash. Radiotherapy (45 Gy over 25 fractions) was administered to all 17 patients and was interrupted for 1 week in only 1 patient because he developed grade 3 neutropenia. All patients except 2 received planned cisplatin dose (30 mg/m^2^ administered intravenously for 2 h on days 1, 8, 15, 22, and 29). The two patients received 80% of their planned doses because they developed grade 3 nausea, neutropenia, and leukopenia. The dose of paclitaxel (50 mg/m^2^, administered intravenously for 1 h on days 1, 8, 15, 22, and 29) was adjusted in 15 patients because they developed hematological toxicity, in accordance with the protocol’s dose modification criteria. Table 2 presents a summary of the neo-NTP-CRT treatment protocol.

**Table 2:** The completeness of neo-NTP-CRT protocol treatment.

Treatment	No. of cycles or fractions		Total dose		
	Planned	Received (mean [range])	Planned	Received (mean [range])	Received in % of planned total dose (mean [range])
Nivolumab	4	3.8 [2~4]	960 mg	918 mg [480~960]	96% [50–100]
Paclitaxel	5	4.1 [3~5]	250 mg/m^2^	188 mg/m^2^ [120~250]	75% [48–100]
Cisplatin	5	4.9 [4~5]	150 mg/m^2^	146 mg/m^2^ [120~150]	97% [75–100]
Radiation	25	25 [25~25]	45 Gy	45 Gy [45~45]	100% [100–100]

#### Surgery

To comply with the proposed protocol, radical surgery was scheduled 6 to 8 weeks after the completion of neo-NTP-CRT, and it was designated as feasible within 12 weeks after neo-NTP-CRT. A total of 14 patients underwent minimally invasive esophagectomy with 2- or 3-field lymph node dissection within 12 weeks after completing their neo-NTP-CRT regimen. Of the 3 patients who did not undergo surgery, 1 developed a progressive disease with an esophagopulmonary fistula after neo-NTP-CRT and received palliative chemotherapy, 1 developed a progressive disease with femoral bone metastasis after neo-NTP-CRT and received palliative radiotherapy over the femoral bone metastasis and a second round of CRT over primary esophageal tumour and regional lymph nodes with a cumulative RT dose of 66 Gy, and 1 had clinical partial response to neo-NTP-CRT but developed pulmonary emboli after neo-NTP-CRT, which was considered inoperable, and also received a second round of CRT over primary esophageal tumour and regional lymph nodes with a cumulative RT dose of 66 Gy. None of the patients died within 1 month after surgery. Table 3 provides a summary of the intervals between neo-NTP-CRT completion and surgery, surgical complications with Clavien-Dindo classification, intensive care unit stay, and hospital stay. A total of 2 patients developed anastomotic leakages, which necessitated surgical interventions. Of these patients, 1 could not recover and died 263 days after surgery.

**Table 3:** Summary of surgery following neo-NTP-CRT.

Parameters related to surgical intervention	Median duration (range) or Other descriptions
Interval from neo-NTP-CRT completion to surgery	65 days (35~82)
ICU stay after surgery	5 days (1~22)
Hospital stay after surgery	16 days (10~263)
Clavien-Dindo Classification of surgical complications	
Grade II	1 (pneumonitis)
Grade IIIa	1 (subclavian artery rupture)
Grade IIIb	1 (anastomotic leakage)
Grade V	1 (anastomotic leakage)

#### Response to neo-NTP-CRT

Patient response was evaluated 3 to 4 weeks after the completion of neo-NTP-CRT. This evaluation involved EUS, FDG-PET, and CT scan. In total, 3 patients demonstrated a complete clinical response, 11 patients demonstrated a partial clinical response, and 3 patients exhibited clinical disease progression, including 2 with locoregional progression and 1 with distant metastasis. One of the two patients with locoregional progression was still considered operable and received esophagectomy; the other two patients with progressive disease did not receive esophagectomy.

Table 4 presents a summary of the pathologic responses observed in the surgical specimens after the completion of neo-NTP-CRT. Of 14 patients who underwent an esophagectomy following neo-NTP-CRT, 13 achieved R0 resection, and 1 had a circumferential margin involved (≤1 mm). A total of 4 patients exhibited pCR; thus, the criterion (of there being 5 patients or more) for entering the second stage of the study was not met. The pCR rate in the intent-to-treat population was 24%, and the pCR rate in the esophagectomy population was 29%. The post-neo-NTP-CRT pathologic stages were ypStage I in 6 patients, ypStage II in 1 patient, ypStage IIIA in 6 patients, and ypStage IIIB in 1 patient. The tumour regression grades were 1a, 1b, 2, and 3 in 5, 3, 2, and 4 patients, respectively.

**Table 4:** Summary of pathological response following neo-NTP-CRT.

		No.	%
Total patient number		14	100
Pathological stage*			
	ypT0N0	4	29
	ypT0N1	1	7
	ypT1N0	1	7
	ypT1N1	2	14
	ypT2N0	1	7
	ypT2N1	3	21
	ypT3N0	1	7
	ypT4aN0	1	7
Resection margin involved			
	Proximal	0	0
	Distal	0	0
	Circumferential	1	0
Tumor regression grade^#^			
	1a	5	36
	1b	3	21
	2	2	14
	3	4	29

*Pathological stage according to AJCC 8th ed.

^#^Tumor response grade according to Brucher et al., 2006; Chang et al., 2008; Chirieac et al., 2005.

#### Survival

**Fig. 1:**
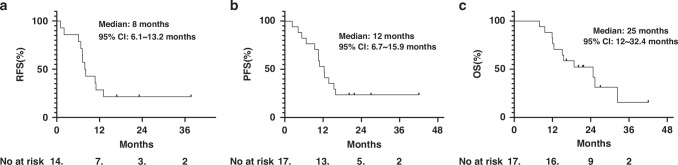
The survival curves of the patients who received neo-NTP-CRT. **a** Median RFS is 8 months; 1-year RFS is 30%; 2-year RFS is 21%. **b** Median PFS is 12 months; 1-year PFS is 55%; 2-year PFS is 24%. **c** Median OS is 25 months; 1-year OS is 75%; 2-year OS is 52%.

After the database was locked on August 18, 2024 (3 years and 7 months after the first patient was enroled), of 14 patients who underwent surgery, 11 had recurrent disease and died, and 3 were followed up. Of the 3 patients who did not undergo surgery, 1 developed an esophagopulmonary fistula and died. The median follow-up period was 27 months, with a median RFS of 8 months (95% confidence interval (CI): 6.1~13.2 months), a median PFS of 12 months (95% CI: 6.7~15.9 months), and a median OS of 25 months (95% CI: 12~32.4 months). All patients died of cancer. Figure 1 depicts the curves of RFS, PFS, and OS.

In terms of recurrent patterns, of the 11 patients who experienced recurrence after surgery, 6 had only locoregional recurrence within the previously irradiated regions, 4 had only distant recurrence, including 3 patients with lung metastasis, 1 patient with bilateral malignant pleural effusion, and 1 patient had both locoregional and distant recurrences with pericardium, bone, and distant lymph node metastasis. The median OS after recurrence was 5 months.

#### Treatment toxicity

Table 5 presents a summary of the incidence of toxicity. Treatment emergent adverse events (TEAEs) were observed in 15 patients (88%) while they were receiving their neo-NTP-CRT, with 14 patients demonstrating CRT-related toxicity and 8 patients demonstrating immune-related toxicity. TEAEs of grade 3 or higher were observed in 4 patients with grade 3 CRT-related leukopenia. The most common grade 1 or 2 CRT-related TEAE was bone marrow toxicity, with 9 patients developing leucopenia, 9 patients developing neutropenia, 3 patients developing anaemia, and 6 patients developing thrombocytopenia. The most common grade 1 or 2 immune-related TEAEs were skin rash in 6 patients and colitis, hypothyroidism, and adrenal insufficiency in 1 patient (Table 6). No patients had grade 3 or higher immune-related TEAEs, and no patients had ever received systemic corticosteroid for immune-related TEAEs.

**Table 5:** Summary of adverse events of neo-NTP-CRT, shown in patient number (percentage) [Total patient number = 17].

	Any grade	Grade 3	Grade 4
Leukopenia	13 (76%)	4 (24%)	0
Neutropenia	10 (59%)	1 (6%)	0
Thrombocytopenia	6 (35%)	0	0
Skin rash	6 (35%)	0	0
Anemia	3 (18%)	0	0
Nausea/ vomiting	1 (6%)	1 (6%)	0
Kidney	1 (6%)	0	0
Hyponatremia	1 (6%)	0	0
Colitis	1 (6%)	0	0
Thyroid	1 (6%)	0	0
Adrenal gland	1 (6%)	0	0
Neutropenic fever	0	0	0
Hepatitis	0	0	0
Pneumonitis	0	0	0

[Note] The highest grades (according to CTC-AE version 5) each individual subject experienced are presented.

**Table 6:** Immune-related adverse events of neo-NTP-CRT, shown in patient number (percentage) [Total patient number = 17].

	Any grade	Grade 3	Grade 4
Skin rash	6 (35%)	0	0
Colitis	1 (6%)	0	0
Hypothyroidism	1 (6%)	0	0
Adrenal insufficiency	1 (6%)	0	0
Pneumonitis	0	0	0
Hepatitis	0	0	0
Infusion-related reaction	0	0	0
Arthritis	0	0	0
Kidney	0	0	0

[Note] The highest grades (according to CTC-AE version 5) each individual subject experienced are presented.

#### Biomarker study

**Fig. 2:**
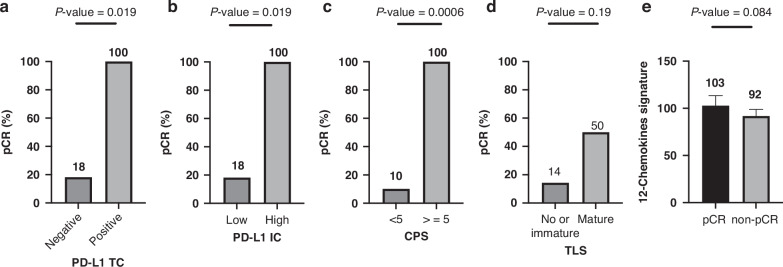
The association of various immune-realted biomakers with pCR. **a** PD-L1 TC (*n* = 14), **b** PD-L1 IC (*n* = 14), **c** CPS (*n* = 14), **d** mature TLS (*n* = 14), and **e** 12-chemokine signature (*n* = 10).

**Fig. 3:**
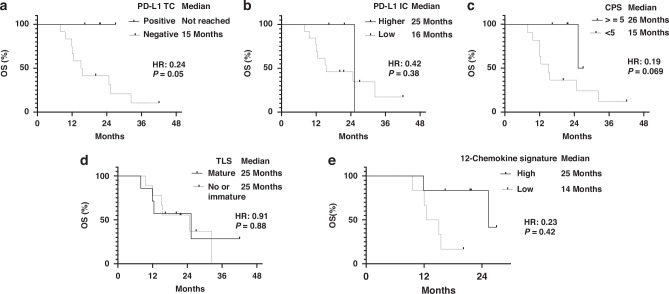
The association of various immune-related biomarkers with OS. **a** PD-L1 TC (*n* = 16), **b** PD-L1 IC (*n* = 16), **c** CPS (*n* = 16), **d** mature TLS (*n* = 16), and **e** 12-chemokine signature (*n* = 12).

**Fig. 4:**
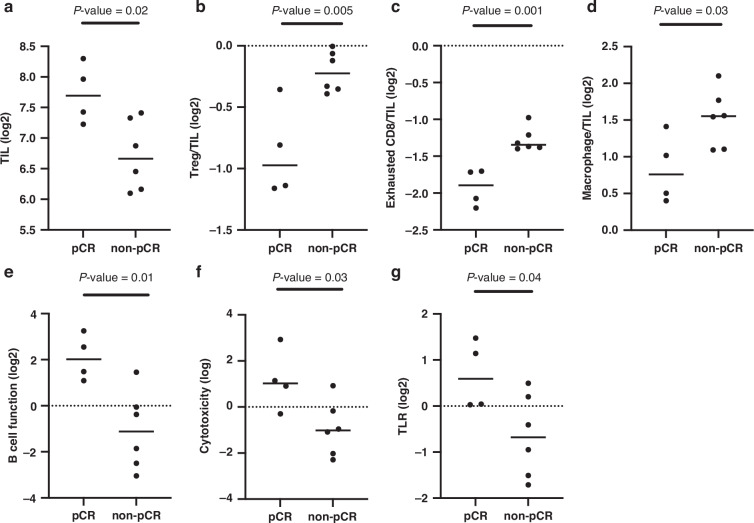
The comparisons of the expression levels of various immune-related genes (*n* = 10) between patients with pCR and those with non-pCR. **a** Tumor-infiltrating lymphocyte (TIL), **b** Regulatory T cell (Treg)/TIL, **c** exhausted CD8/TIL, **d** macrophage/TIL, **e** B cell function, **f** cytotoxicity, and **g** Toll-like receptor (TLR).

The rate of pCR significantly increased in patients with positive PD-L1 TCs (*n* = 14, 100% vs 18%, *P* = 0.019), high PD-L1 ICs (*n* = 14, 100% vs 18%, *P* = 0.019), and CPS ≥ 5 (*n* = 14, 100% vs 10%, *P* = 0.0006) and had a trend of increase in patients with mature TLSs (*n* = 14, 50% vs 14%, *P* = 0.19). Compared with patients without pCR, those with pCR had a trend of higher 12-chemokine TLS signature (*n* = 10, *P* = 0.084; Fig. 2). Patients with positive PD-L1 TCs (*n* = 16, median: not reached vs 15 months, hazard ratio: 0.24, *P* = 0.05) and CPS ≥ 5 (*n* = 16, median: 26 vs 15 months, hazard ratio: 0.19, *P* = 0.069) had a trend of increased OS, and patients with high PD-L1 ICs (*n* = 16, median: 25 vs 16 months, hazard ratio: 0.42, *P* = 0.38) had numerically increased OS. No significant association was observed between mature TLSs and OS (*n* = 16, median: 25 vs 25 months, hazard ratio: 0.91, *P* = 0.88); however, patients with a higher 12-chemokine TLS signature (median cutoff) had significantly longer OS (*n* = 12, median: 25 vs 14 months, hazard ratio: 0.23, *P* = 0.042; Fig. 3). An analysis of immune-related gene expression (*n* = 10) revealed that patients with pCR had a significantly increased number of tumour-infiltrating lymphocytes (*P* = 0.02) but a significantly decreased number of regulatory T cells (*P* = 0.005), exhausted CD8 T cells (*P* = 0.001), and macrophages (*P* = 0.03; Fig. 4). They also exhibited significantly increased B-cell function (*P* = 0.01), cytotoxicity function (*P* = 0.03), and Toll-like receptor function (*P* = 0.04; Fig. 4).

## Discussion

Overall, the primary endpoint was not met in our clinical trial. Our pCR rate (24%) did not demonstrate the potential of our protocol to increase the rate of pCR from 25% to 45%. In addition, our median PFS and OS did not exceed those reported in our previous study (12 vs 14 months for PFS, 25 vs 22 months for OS) [29]. The neoadjuvant therapy with neo-NTP-CRT protocol was associated with acceptable toxicity profiles without new safety signal. A total of 4 patients experienced major surgical adverse events after surgery, including 2 with anastomotic leakages, which necessitated surgical intervention. Overall, our results, based on a relatively small sample size, did not support the combination of an anti-PD-1 antibody with neoadjuvant CRT as a promising treatment strategy for patients with locally advanced ESCC.

Two clinical trials investigating pembrolizumab, an anti-PD-1 monoclonal antibody approved for the treatment of recurrent or metastatic ESCC, in combination with neoadjuvant CRT for the treatment of patients with locally advanced ESCC reported conflicting results regarding the rate of pCR. In the trial conducted by Li et al. [37] in China, 20 patients received pembrolizumab in combination with neoadjuvant CRT using CROSS regimen [2]. Of these patients, 1 died while undergoing CRT. A total of 18 patients underwent an esophagectomy, and 10 patients achieving pCR, with a pCR rate of 50%. In the trial conducted by Park et al. [38] in South Korea, 16 patients received paclitaxel and carboplatin-based neoadjuvant CRT involving pembrolizumab. All patients underwent an esophagectomy, but only 1 patient achieved pCR, with a pCR rate 6%. Of all patients, 2 died from surgical complications associated with acute respiratory distress syndrome or pneumonitis. The wide-range of pCR rates, ranging from 6% to 50%, reported in the above-mentioned clinical trials investigating the addition of anti-PD-1 to neoadjuvant CRT may result from small sample size, patient selection bias, and variable neoadjuvant treatments. There are currently 4 ongoing randomized phase 3 trials investigating the benefit of adding anti-PD1 antibodies to neoadjuvant CRT. Two, NCT03957590 and NCT04973306, used tislelizumab, and the other two, NCT05244798 and NCT05357846, used sintilimab. The results may clarify whether this treatment strategy is feasible and beneficial.

To determine the role of immunotherapy in neoadjuvant treatment, various clinical trials, predominantly conducted in China, have explored the effects of integrating anti-PD1 antibodies into the neoadjuvant chemotherapy for patients with locally advanced ESCC [39–42]. In these phase II single-arm trials, the rate of pCR was reported to range between 15% and 40%, which is higher than the historical control of 2% for neoadjuvant chemotherapy using cisplatin combined with either 5-fluorouracil or paclitaxel. A multicenter, randomized, open-label phase 3 trial comparing neoadjuvant camrelizumab plus chemotherapy with neoadjuvant chemotherapy alone was published in 2024 [43]. A total of 391 patients were randomized in a 1:1:1 ratio to two cycles of camrelizumab plus albumin-bound paclitaxel and cisplatin, camrelizumab plus paclitaxel and cisplatin, and paclitaxel with cisplatin. In the intention-to-treat population, both arms with camrelizumab showed significantly higher pCR rate than chemotherapy alone (28% and 15% vs 4.7%, *P* < 0.0001 and *P* = 0.0034, respectively).

Although preclinical research has suggested that radiotherapy has a synergistic effect on immunotherapy [44], other studies have indicated that radiotherapy affects anticancer immunity. Some of these studies have reported that radiotherapy has a detrimental effect on the antitumor immunity generated from the drainage lymph nodes of the tumour. For example, using a head and neck squamous cell carcinoma mouse model, Saddawi-Konefka et al. [45] discovered that the ablation of drainage lymph nodes by radiation mitigated the tumour’s response to ICIs with decreasing OS. In their mechanistic study, they reported that radiation eliminated conventional type I dendritic cells in the drainage lymph nodes, which was essential for the patients’ response to ICIs. Using a similar mouse model, Darragh et al. [46] discovered that exposing drainage lymph nodes to radiation systemically attenuated the radiation-induced immune response of patients by decreasing their antigen-specific T cells and epitope spreading, thereby promoting the growth of local and metastatic tumours. Overall, these preclinical studies provide mechanistic insights into the lack of improvement in the antitumor efficacy of neoadjuvant CRT when anti-PD-1 immunotherapy is added, as observed in the present phase II study. In a retrospective study published in 2025, higher estimated dose of radiation to immune cells was found to associate with lower lymphocyte nadir during CRT and worse disease-free survival and OS in a cohort of 182 locally advanced ESCC patients who received neoadjuvant CRT followed by esophagectomy [47]. In another retrospective study of 512 patients from 2 prospective clinical trials of definitive CRT, higher radiation dose to tumour drainage lymph nodes was found to associate with lower B cells and activated CD8 T cells in peripheral blood, and worse local recurrence-free survival and distant metastasis-free survival [48]. Overall, these two clinical studies revealed that radiation over local or systemic immune systems may have impact on anti-tumour immunity and patients’ prognosis. Further research is required to confirm our clinical observations and the underlying mechanisms.

In this study, we discovered that patients with high PD-L1 expression in primary esophageal tumours had significantly higher pCR rate than those with low PD-L1 expression, suggesting that PD-L1 expression may serve as a biomarker of patient responses to neo-NTP-CRT. Our observation is in line with previous studies which reported that PD-L1 expression of either TCs or CPS was associated with a survival benefit from anti-PD1 ICIs in multiple randomized phase III trials of recurrent or metastatic ESCC [6, 7, 11, 12]. In our study, we also found that although mature TLSs, as defined by immunohistochemistry, failed to reflect patient responses to neo-NTP-CRT, the 12-chemokine TLS signature was significantly associated with OS. Multiple retrospective studies have indicated that TLSs influence the efficacy of ICIs against various types of cancer [36, 49–53]. In one of these studies, which included a retrospective ESCC cohort, Hayashi et al. [49] reported that the density and maturity of TLSs in resected specimens were significantly associated with treatment response and OS in 34 patients with recurrent ESCC who were treated with anti-PD1 ICIs. Despite our small sample size, our findings indicate that PD-L1 expression and mature TLSs may aid in the selection of patients who may benefit from adding anti-PD1 ICIs to their conventional neoadjuvant CRT.

Our study has several limitations. First and foremost, this is a single-arm phase II trial with a relatively small sample size. We designed the trial with Simon’s 2-stage design with 17 patients enroled in the first stage and 41 patients in total. Only 4 patients achieved pCR in the first stage; therefore, it was unable to fulfill the criteria, 5 patients, to enter the second stage. Simon’s 2 stage design can limit the number of patients from receiving ineffective treatment; however, it also limits the probability of rejecting the null hypothesis and the potential of generating new hypothesis to further develop the novel treatment. Based on the 17 patients enroled in the trial, we are still at risk of reaching wrong conclusion. Secondly, the trial was designed before the publication of the results of CHECKMATE 577; therefore, adjuvant nivolumab was not added to the treatment protocol of the trial, and one of the patients received adjuvant nivolumab. Having adjuvant nivolumab in the trial protocol might influence survival outcomes, but would not change the result of the primary outcome, pCR, of the trial. Thirdly, in the biomarker study, endoscope-biopsied tumour specimens were used and may not represent the whole tumours, especially for TLS. Besides, we did not have an independent cohort to validate the results.

In conclusion, the current phased II study demonstrated the feasibility and safety of neoadjuvant immunochemoradiotherapy using the combination of nivolumab, cisplatin, and paclitaxel in patients with locally advanced ESCC. A modest rate of pCR did not support such an approach have a positive impact on outcomes of these patients; however, a subgroup of patients with high expression of PD-L1 in the primary ESCC tissues might have better outcomes from the neoadjuvant immunochemoradiotherapy.

## Data Availability

Raw data are available from the corresponding author upon reasonable request.
